# Interrater reliability in the temporal quantitative analysis of oropharyngeal swallowing using a specific software

**DOI:** 10.1590/2317-1782/20212020389

**Published:** 2021-10-25

**Authors:** Paula Cristina Cola, Thaís Coelho Alves, Ana Rita Gatto, Claudio José Rubira, André Augusto Spadotto, Roberta Gonçalves da Silva

**Affiliations:** 1 Departamento de Medicina, Universidade de Marília – UNIMAR - Marília (SP), Brasil; 2 Laboratório de Pesquisa e Reabilitação em Disfagia, Departamento de Fonoaudiologia, Universidade Estadual Paulista – UNESP - Marília (SP), Brasil

**Keywords:** Swallowing, Time, Software, Deglutition Disorders, Reliability, Deglutição, Tempo, Software, Transtornos de Deglutição, Concordância

## Abstract

**Purpose:**

This study aims to analyze inter-judge reliability by measuring a few temporal parameters of swallowing using a specific software.

**Methods:**

Six databases that include the results of reliability tests performed in prior studies by the present research group were employed. The datasets were obtained using the same measurement method and include data obtained based on puree (level 3) consistency and liquid (level 0) consistency according to the International Dysphagia Diet Standardization Initiative. In this study, the reliabilities corresponding to the total oral transit time (TOTT), initiation of the pharyngeal response time (PRT), and the pharyngeal transit time (PTT) were measured using the same software; the evaluations performed by a single rater for all datasets were used as the benchmark, and evaluations performed by new raters for each dataset were also included. The intra-class correlation coefficient (ICC) with a 95% confidence interval was employed.

**Results:**

A total of 244 videofluoroscopic swallowing study images were analyzed. In all analyses, the ICCs were >0,75 and showed excellent agreement between the senior and junior raters. The TOTT for level 3 showed ICCs from 0.936 to 1.000 and that for level 0 showed ICCs from 0.997 to 1.000. Further, the PRT showed ICCs from 0.916 to 1.000 for level 3 and from 0.978 to 1.000 for level 0. The PTT showed ICCs from 0.848 to 1.000 for level 3 and from 0.984 to 1.000 for level 0.

**Conclusion:**

The reliabilities obtained using this specific software for the TOTT, PRT, and PTT showed excellent agreement.

## RESUMO

**Objetivo:**

analisar a confiabilidade inter-juízes por meio da mensuração de alguns parâmetros temporais da deglutição por meio de um software específico.

**Método:**

Foram utilizados seis bancos de dados que incluem os resultados dos testes de confiabilidade realizados em estudos anteriores do presente grupo de pesquisa. Os bancos de dados foram obtidos usando o mesmo método de medição e incluem dados obtidos na consistência purê (nível 3) e líquida (nível 0). Neste estudo, as confiabilidades correspondentes ao tempo de trânsito oral total (TTOT), tempo de início de resposta faríngea (IRF) e tempo de trânsito faríngeo (TTF) mensuradas no mesmo software; as avaliações realizadas por um único avaliador para todos os bancos de dados foram usadas como referência, e as avaliações realizadas por novos avaliadores para cada banco de dados também foram incluídas. Foi utilizado o coeficiente de correlação intraclasse (ICC) com intervalo de confiança de 95%.

**Resultados:**

Foram analisadas 244 imagens de estudos videofluoroscópicos da deglutição. Em todas as análises, os ICCs foram >0,75, mostrando excelente concordância entre avaliadores. O TTOT do nível 3 apresentou ICCs de 0,936 a 1.000 e o do nível 0 apresentou ICCs de 0,997 a 1,000. Além disso, o IRF mostrou ICCs de 0,916 a 1,000 para o nível 3 e de 0,978 a 1,000 para o nível 0. O TTF mostrou ICCs de 0,848 a 1,000 para o nível 3 e de 0,984 a 1,000 para o nível 0.

**Conclusão:**

As confiabilidades obtidas com este software específico para o TTOT, IRF e TTF mostraram excelente concordância.

## INTRODUCTION

The interrater reliability in videofluoroscopic swallowing studies (VFSS) has been investigated to decrease the variability of findings related to swallowing, including the detection and interpretation of posterior oral spillage, penetration, aspiration, and the use of protocols and scales^(1,2)^. With regard to the standardization of the VFSS procedure, the results of studies entailing temporal measures and the lack of interrater reliability are not concordant.

One study involving qualitative parameters aimed to determine whether directed search or free search can provide a substantially reliable interpretation of VFSS results. The results of the study suggested that for experienced speech language pathologists, a free search may be optimal for identifying aspiration, penetration, and other salient symptoms and attributes of dysphagia. Although the interrater reliability results obtained using a free search appear promising, further research is needed to determine the manner in which the search method impacts detection, decision making, and accuracy with regard to VFSS measures^(3)^.

VFSS serve as a crucial method to obtain knowledge regarding swallowing disorders. In this regard, one study assessed the interrater reliability of videofluoroscopy for swallowing evaluation based on qualitative and quantitative parameters. The results showed that the interpretation of videofluoroscopy results will continue to entail an extensive interrater variability until a greater number of specific diagnostic variables are systematically evaluated. Only findings regarding aspiration are well defined, and such findings are consequently diagnosed with high interobserver agreement^(4)^.

The results of the temporal quantitative analysis of oropharyngeal swallowing can vary substantially depending on the definitions of parameters, the analysis software used, and the expertise of the rater^(5-7)^. A systematic review and meta-analysis of recent studies showed that current evidence regarding temporal measures of swallowing combined with penetration and aspiration in patients with dysphagia is limited owing to heterogeneity^(8)^. The authors suggest that performing continued research with a reproducible standard and approach for measuring temporal characteristics is crucial.

A possibly adequate technique should be capable of prolonged measurements, as well as slow motion, frame by frame, and software-based measurements. One study compared the use of a timer and that of a software to perform the quantitative measurement of pharyngeal transit time. Notably, different mean pharyngeal transit time values were obtained using the timer and the software, and the authors reported the need for accurate tools and specific programs^(6)^. Some studies used quantitative temporal measures but did not clearly elucidate all the methods applied^(9-12)^.

Moreover, it is also important to train the raters with regard to the method being investigated. The authors of a report entailing the fiberoptic endoscopic evaluation of swallowing (FEES) and measurement of the whiteout time reported that they trained raters in their study via practical and theoretical training for 12 hours. Particularly, the training was conducted in a frame-by-frame manner and under the supervision of a senior member. The practical training in this study was classified into analyses of the images by individual raters as well as by groups under supervision^(13)^.

Although studies on the qualitative and quantitative analysis of swallowing have been reported with varied operational definitions, methods, and results, there are only a few studies on the training of raters and their interrater reliability for temporal measurements. Therefore, this study aimed to analyze the interrater reliability for the measurement of temporal quantitative analysis parameters in post-stroke individuals using specific software.

## METHODS

This study was approved by the Human Research Ethics Committee of the institution, under protocol 2.671.11/2018. However, all other studies had their own ethics committee and an informed consent term for the participants.

### Design of study

This was a retrospective clinical study. Particularly, this study included six datasets obtained from six prior studies conducted in the same research center. Each of these six studies^(14-18)^ performed quantitative temporal measurements using one expert rater (senior) and a junior rater. The swallowing exhibited by patients after stroke, both genders was analyzed; the age range of the patients was 50-80 years. Each patient was trial equal volumes of puree and liquid bolus using a spoon. The patients were instructed to swallow immediately after the placement of the bolus in the oral cavity.

### Characteristic of raters and training

All raters in this study were speech language pathologists who specialized in oropharyngeal dysphagia and had substantial experience in performing instrumental examinations, especially VFSS. The experience of the raters ranged between 5-10 years. The first rater (rater 1- senior) was involved in the development of the software used in the present study and was considered to have the most expertise with regard to the method. Four other raters (juniors) were separately trained for approximately 6 hours over three days by rater 1 to quantitatively analyze the VFSS results.

The training method consisted primarily of the theory of the temporal quantitative analysis of oropharyngeal swallowing in the VFSS images, in the presentation of the software and its prior publication in the literature^(6)^. This software, although published, is not commercialized and at the moment, it remains in use with the research group and/or partner researchers.

All juniors raters were trained to use the software and its tools correctly, as well as to delimit the anatomical points used as parameters for 2 hours. Subsequently, the juniors and the senior rater performed the joint practice in the software, analyzing each parameter of this study in approximately 5 VFSS of each consistency. At this time, they discussed doubts about the operation of the software and analysis of the parameters for 4 hours. Then, independently, each junior rater evaluated each parameter of this study in approximately 5 other exams of each consistency. The senior rater also independently and blindly to the results of the analysis of the juniors raters performed the analysis of the same VFSS for the same parameters. At the end of the analysis, the senior rater met with the junior rater and they checked the results. If there was a disagreement between these two raters, the senior would restart the training of the junior judge. If not, both were able to analyze all the images included for each study.

### Characteristic of VFSS

A Prestilix seriographer, model 1600X (1000 MA, 130 KV–GE), operated via remote control was used. The images were transmitted to a video monitor (Sony, model PVM-95E). All of the VFSS videos captured the region from the oral cavity to the esophagus at an acquisition rate of 29.97 frames per second; thus, the position of the bolus could be assessed approximately every 33 milliseconds. Further, for the first trial, puree and/or liquids with consistencies of 3 and 0, respectively, in accordance with the International Dysphagia Diet Standardization Initiative (IDSSI)^(19)^ were administered once with a spoon as single 5 ml volumes. For the exam, all of the puree and liquid samples were prepared with the same protocol, that is, using only a food thickener, water, and barium for puree and using only water and barium for liquids.

### Protocol for measurement

Specific software developed by our research group^(6)^ was employed to measure the quantitative temporal parameters of swallowing based on VFSS videos with 29.97 frames per second. This software analyzes the VFSS images in a frame-by-frame and provides the time in milliseconds. Although the rater needs to be trained in using the tools of the software, we emphasize that it is easy to use.

In this study, three parameters of the oropharyngeal swallowing time were analyzed: Total oral transit time (TOTT), pharyngeal response time (PRT), and pharyngeal transit time (PTT)^(20-23)^. Note that were different database; therefore, each study evaluated one or two time parameters.

The TOTT was defined as the interval in milliseconds, between the first image showing the food inside the oral cavity and the first frame showing the proximal part of the food bolus in the final region of the hard palate and the beginning of the soft palate, without the proximal part of the food bolus exceeding the lower ramus of the mandible, as proposed by Logemann et al.^(21)^ and adapted by Gatto et al.^(22)^.

The PRT was defined as the interval, in milliseconds, from the frame showing the proximal part of the food bolus at the final region of the hard palate and the beginning of the soft palate, where the lower rim of the mandible crosses the tongue base, to the first frame showing the laryngeal elevation^(21)^.

Further, the PTT was considered as the interval between the time when the bolus was in the final region of the hard palate and the beginning of the soft palate, forming an angle with the mandibular branch and the base of the tongue, and the time when pharyngeal phase swallowing ended, that is, the moment when the bolus passed through the upper esophageal sphincter^(23,24)^.

### Statistical analysis

Statistical analysis was performed using the Statistical Package for Social Sciences (SPSS) program, and the adopted significance level was 5%. After the measurement of each quantitative parameter by the raters, the mean values were used to determine the intra-class correlation coefficient (ICC), with a 95% confidence interval. The classification values of the ICC were used according to Cicchetti^(25)^. Only the Interrater reliability were performed due to the fact that the senior rater was present in the development of the software, the longer experience in the temporal quantitative analysis of oropharyngeal swallowing and previous publications. The analysis of the level of interrater reliability was performed for the images obtained from each study.

## RESULTS

A total of 244 swallows images that were evaluated by rater 1 and raters 2, 3, 4, and 5 analyzed, respectively, 61 swallows images from study 1, 22 from study 2, 28 from study 3, and 73 from study 4. Rater 1 and 3 evaluated the images obtained via studies 5 and 6, with 30 swallows images corresponding to each study. For all the studies, the ICCs were >0,75, showing excellent agreement.

Table 1 shows the ICC obtained based on the TOTT for level 3 and level 0. For level 3, the TOTT showed ICCs from 0.936 to 1.000. For level 0, ICCs from 0.997 to 1.000 were obtained.

**Table 1:** Interrater reliability obtained using the intraclass correlation coefficient (ICC) for the total oral transit time for each study for level 3 and level 0

TOTT		N	ICC	Inferior limit	Upper limit	*P* value
	*Level 3*					
*Study 1*		61	1.000	.999	1.000	.000
*Study 2*		22	1.000	.999	1.000	.000
*Study 3*		28	.970	.936	.986	.000
	*Level 0*					
Study 2		21	.999	.997	1.000	.000

Intraclass correlation coefficient (ICC) with a 95% confidence interval

**Caption:** N: number of swallows; TOTT: Total oral transit time

Table 2 shows the ICCs obtained based on the PRT for level 3 and level 0. For level 3, the PRT showed ICCs from 0.916 to 1.000. For level 0, ICCs from 0.978 to 1.000 were obtained.

**Table 2:** Interrater reliability obtained using the intraclass correlation coefficient (ICC) for pharyngeal response time (PRT) for each study for level 3 and level 0

PRT		N	ICC	Inferior limit	Upper limit	*P* value
	*Level 3*					
*Study 1*		61	.998	.996	.999	.000
*Study 2*		22	1.000	.999	1.000	.000
*Study 3*		28	.960	.916	.981	.000
*Study 4*		73	.997	.995	.998	.000
	*Level 0*					
Study 2		21	.991	.978	.996	.000
Study 4		22	1.000	.999	1.000	.000

Intraclass correlation coefficient (ICC) with a 95% confidence interval

**Caption:** N: number of swallows; PTT: Pharyngeal response time

Table 3 shows the intraclass correlation coefficient (ICC) obtained based on the PTT for level 3 and level 0. For level 3, the PTT showed ICCs from 0.848 to 1.000. For level 0, ICC values ranging from 0.984 to 1.000 were obtained.

**Table 3:** Interrater reliability obtained using the intraclass correlation coefficient (ICC) for pharyngeal transit time (PTT) for each study for level 3 and level 0

PTT		N	ICC	Inferior limit	Upper limit	*P* value
	*Level 3*					
*Study 1*		61	.998	.997	.999	.000
*Study 2*		22	1.000	.999	1.000	.000
*Study 5*		30	.953	.848	.982	.000
*Study 6*		30	.998	.996	.999	.000
	*Level 0*					
Study 2		21	.993	.984	.997	.000

Intraclass correlation coefficient (ICC) with a 95% confidence interval

**Caption:** N: number of swallows; PTT: Pharyngeal transit time

## DISCUSSION

Systematic review studies on quantitative temporal analysis of swallowing concluded that there is no consensus on methodological practices in this area, mainly owing to variations in the definition of parameters, variations in training, low reliability between judges, software used and study design^(7,8,26)^.

Although an evaluation of the swallowing biomechanics through VFSS is the gold standard, the quality of training of the rater in qualitative or quantitative measurements can always interfere with the analysis. Thus, studies from the past few decades have demonstrated the importance of agreement between raters during the analysis. So far, it is known that training of raters is necessary and, even so, not all of them present an adequate agreement in their analysis^(3,4)^. In addition, discussion between group raters, observation time, quality of the images, and training protocols are factors that influence the level of agreement between analysis^(5,27)^.

Before discussing the results of the reliability of judges from this study, it is essential to reflect on the role of training judges with a survey on the quantitative temporal analysis of swallowing. It is known that inter- and/or intra-judge reliability is necessary to decrease variabilities in detection, interpretation, and clinical decision-making for patients with oropharyngeal dysphagia. A systematic review of the literature on reliability in VFSS showed that among the 19 included studies, only a few reported on reliability protocols^(27)^. The authors emphasize that any swallowing study must provide information about training and intra-rater and interrater reliability to allow for an accurate interpretation of the research.

The results of this study showed excellent agreement between the senior and junior raters across all parameters and consistencies of the foods studied, which can be explained by the standardization of terminologies, training following pre-established protocols, training time, and joint analyses. Thus, this is in agreement with several previous studies, which demonstrated that training of judges increased the reliability of the research results^(5,28)^.

In this paper, the protocol used for determining the reliability of raters with characteristics such as specialty of the judges, length of experience, expertise in the subject, and training in the method used, which are important factors for increasing reliability, is also described. In addition, as this is a research study with a quantitative analysis of oropharyngeal swallowing, training in the software used and previous definitions of the measurements are necessary. In the studies included in the systematic review, some did not include descriptions of elements such as the judges’ experience, reliability protocols, and whether the judges were blind to the ratings of other judges or to the subjects' clinical conditions^(27)^.

Another issue to be discussed is the operational definitions used by studies for describing the analyzed parameters. There are divergences in these definitions, with some authors who determine the beginning or the end of each one by means of anatomical aspects^(18,24)^ and others who determine it by physiological means^(29,30)^. In addition to a lack of standardization that compromises the comparison between surveys, these discrepancies in terminology and analyses lead to different results and hinder the reproducibility of any results of this method.

Unfortunately, one of the limiting factors of this study is that the software used is not commercialized yet. Although the software has already been made available to other research groups, studies comparing these different centers have not yet been carried out. For future research, it is considered the analysis of reliability between different centers.

Finally, it should be considered that the excellent reliability found in this study occurred in the context of the experience of the research group in handling the parameters and software used.

## CONCLUSION

The reliabilities obtained for the TOTT, PRT, and PTT using the specific software employed in this study were excellent.
